# Mitochondrial Dysfunction in Neurodegenerative Diseases

**DOI:** 10.3390/cells14040276

**Published:** 2025-02-13

**Authors:** Han-Mo Yang

**Affiliations:** Division of Cardiology, Department of Internal Medicine, Seoul National University Hospital, Seoul 03080, Republic of Korea; hanname@gmail.com

**Keywords:** mitochondrial dysfunction, neurodegenerative disease, oxidative stress, mitochondrial dynamics

## Abstract

Mitochondrial dysfunction represents a pivotal characteristic of numerous neurodegenerative disorders, including Alzheimer’s disease, Parkinson’s disease, Huntington’s disease, and amyotrophic lateral sclerosis. These conditions, distinguished by unique clinical and pathological features, exhibit shared pathways leading to neuronal damage, all of which are closely associated with mitochondrial dysfunction. The high metabolic requirements of neurons make even minor mitochondrial deficiencies highly impactful, driving oxidative stress, energy deficits, and aberrant protein processing. Growing evidence from genetic, biochemical, and cellular investigations associates impaired electron transport chain activity and disrupted quality-control mechanisms, such as mitophagy, with the initial phases of disease progression. Furthermore, the overproduction of reactive oxygen species and persistent neuroinflammation can establish feedforward cycles that exacerbate neuronal deterioration. Recent clinical research has increasingly focused on interventions aimed at enhancing mitochondrial resilience—through antioxidants, small molecules that modulate the balance of mitochondrial fusion and fission, or gene-based therapeutic strategies. Concurrently, initiatives to identify dependable mitochondrial biomarkers seek to detect pathological changes prior to the manifestation of overt symptoms. By integrating the current body of knowledge, this review emphasizes the critical role of preserving mitochondrial homeostasis as a viable therapeutic approach. It also addresses the complexities of translating these findings into clinical practice and underscores the potential of innovative strategies designed to delay or potentially halt neurodegenerative processes.

## 1. Introduction

Neurodegenerative diseases are widely acknowledged as some of the most significant public health challenges of the modern era, reflecting the cumulative impact of an aging global population [1]. Conditions such as Alzheimer’s disease (AD), Parkinson’s disease (PD), Huntington’s disease (HD), and amyotrophic lateral sclerosis (ALS) each exhibit distinct clinical syndromes and pathological features. AD is characterized by progressive memory loss and the accumulation of amyloid-β (Aβ) plaques alongside tau neurofibrillary tangles, while PD is defined by the loss of dopaminergic neurons in the substantia nigra pars compacta and the presence of Lewy bodies primarily composed of α-synuclein [2,3]. Huntington’s disease results from a CAG sequence expansion in the HTT gene, leading to a mutant huntingtin protein with toxic polyglutamine tracts, whereas ALS involves the degeneration of upper and lower motor neurons with diverse genetic underpinnings [4,5,6].

Despite these differences, a common theme across all four disorders is mitochondrial dysfunction. Early research in Parkinson’s disease demonstrated that the neurotoxin 1-methyl-4-phenyl-1,2,3,6-tetrahydropyridine (MPTP) induces parkinsonism by inhibiting complex I of the electron transport chain (ETC), underscoring the susceptibility of dopaminergic neurons to mitochondrial damage [7,8]. Subsequent studies in AD, HD, and ALS further supported the idea that impaired energy metabolism, excessive reactive oxygen species (ROS) production, and defective mitochondrial quality control often precede severe neurodegeneration [9,10,11]. A compromised mitochondrial network can exacerbate oxidative stress, disrupt calcium buffering, and mismanage pro-apoptotic signals, thereby accelerating neuronal decline.

Aging itself is associated with accumulated damage to mitochondrial DNA (mtDNA), reduced ETC efficiency, and weakened antioxidant defenses, rendering the aging nervous system particularly vulnerable to metabolic stress [12]. Mitochondria also interact closely with other organelles, most notably the endoplasmic reticulum (ER), to regulate intracellular calcium homeostasis. Emerging evidence highlights the importance of ER–mitochondria contact sites, often referred to as mitochondria-associated membranes (MAMs), in neurodegenerative conditions [13]. Additionally, numerous genetic studies have confirmed that mutations in genes responsible for mitophagy or protein folding/aggregation converge on pathwaysthat impair mitochondrial function or hinder their clearance [14,15].

While targeting mitochondrial dysfunction holds conceptual promise, translating this potential into effective therapies remains a significant challenge. Interventions such as coenzyme Q10 or creatine, which aim to enhance energy supply or reduce ROS, often yield only modest benefits in clinical trials [16]. More advanced strategies have emerged, including modulating the balance of mitochondrial fusion and fission to maintain functional networks, enhancing mitophagy to remove damaged organelles, and employing gene-based approaches to correct or reduce the production of toxic proteins [17,18]. Concurrently, there is considerable interest in identifying biomarkers that reflect mitochondrial health, potentially enabling earlier diagnosis or patient stratification for clinical trials. Techniques ranging from peripheral blood mtDNA analysis to advanced neuroimaging are currently under investigation [19,20].

This review aims to elucidate the principal mechanisms underlying mitochondrial dysfunction across neurodegenerative diseases and to compare how each disorder exploits or exacerbates mitochondrial vulnerabilities. We then explore the current landscape of mitochondrial-targeted therapies, including small-molecule antioxidants, modulators of mitochondrial dynamics, gene therapies, and lifestyle interventions. Additionally, we discuss progress in developing biomarkers capable of capturing mitochondrial decline in vivo. A final section provides an integrative discussion of remaining challenges and future directions in this rapidly evolving field.

By examining the interplay among defective ETC components, oxidative stress, proteostatic failure, and impaired quality-control pathways, we illustrate how multiple distinct diseases converge on a single subcellular battleground. Although curative treatments remain elusive, the accelerating pace of research in mitochondrial biology offers renewed hope for slowing or preventing the devastation caused by these disorders. In synthesizing the multifaceted role of mitochondria, we emphasize that preserving their function is not merely supplementary but central to combating the neurodegenerative cascade.

## 2. Mitochondrial Functions and Quality Control

Mitochondria are double-membrane-bound organelles, and their intricate structure, with an outer mitochondrial membrane (OMM), inner mitochondrial membrane (IMM) folded into cristae, intermembrane space (IMS), and matrix, is crucial for their diverse functions.

### 2.1. Mitochondrial Functions

**Oxidative Phosphorylation (OXPHOS):** The ETC, located in the IMM, is composed of five protein complexes (complex I–V). Complex I (NADH:ubiquinone oxidoreductase or NADH dehydrogenase) accepts electrons from nicotinamide adenine dinucleotide (NADH), which is produced during glycolysis and the Krebs cycle. These electrons are then passed to ubiquinone (coenzyme Q) [21]. Complex II (succinate dehydrogenase) also contributes electrons to the ETC, accepting them from flavian adenine dinucleotide (FADH2) (another product of the Krebs cycle) and transferring them to ubiquinone [21,22]. Ubiquinone then carries the electrons to Complex III (cytochrome bc1 complex). Complex III transfers the electrons to cytochrome c, a small, mobile protein located in the intermembrane space [23]. Cytochrome c then carries the electrons to complex IV (cytochrome c oxidase) [23,24]. Complex IV catalyzes the final step in the ETC, transferring electrons to molecular oxygen (O_2_), which is reduced to water (H_2_O). As electrons move through complexes I, III, and IV, protons (H+) are pumped from the mitochondrial matrix into the intermembrane space, creating an electrochemical gradient—the proton-motive force [25]. This gradient provides the energy for complex V (ATP synthase) to synthesize adenosin triphosphate (ATP) from ADP and inorganic phosphate (Pi). This process is highly efficient and generates the vast majority of cellular ATP [17,21,22,23,24,25]. The efficiency of OXPHOS can be affected by various factors including substrate availability, the integrity of the ETC complexes, and the permeability of the inner mitochondrial membrane [22,23,24].

**Mitochondrial Dynamics:** Mitochondria are not static structures; they continuously undergo fusion and fission, processes collectively known as mitochondrial dynamics. Fusion, mediated by mitofusins (Mfn1 and Mfn2) on the OMM and optic atrophy 1 (OPA1) on the IMM, allows for the mixing of mitochondrial contents (proteins, mtDNA, metabolites) [26]. This mixing helps to compensate for localized defects and maintain a healthy mitochondrial network. It is particularly important under conditions of stress, allowing for complementation between damaged and healthy mitochondria [26,27]. Fission, primarily mediated by Drp1 (dynamin-related protein 1), which is recruited to the OMM by adaptor proteins like fission 1 (Fis1), mitochondrial fission factor (Mff), mitochondrial dynamics protein 49 (MiD49), and MiD51, is necessary for the segregation of damaged portions of mitochondria [26,27,28]. This segregation allows for the selective removal of dysfunctional mitochondria via mitophagy. Fission is also important for mitochondrial distribution throughout the cell, particularly in neurons, where mitochondria need to be transported to distal axons and dendrites to meet local energy demands [26,27,28]. Imbalances in fusion and fission are increasingly recognized as key contributors to neurodegenerative diseases.

**Calcium Homeostasis:** Mitochondria play a crucial role in buffering cytosolic calcium levels [29]. They can rapidly take up large amounts of calcium through the mitochondrial calcium uniporter (MCU) complex, located in the IMM. This uptake is driven by the electrochemical gradient across the IMM. Calcium release from mitochondria occurs through various mechanisms, including the mitochondrial permeability transition pore (mPTP), the Na^+^/Ca^2+^ exchanger (NCLX), and the H^+^/Ca^2+^ exchanger [30]. This tightly regulated calcium handling is essential for numerous cellular processes, including synaptic transmission, muscle contraction, and enzyme activation. Mitochondrial calcium overload, however, can trigger the opening of the mPTP, leading to mitochondrial swelling, release of apoptotic factors, and ultimately, cell death [29,30]. Mfn2 also plays a specific role in calcium homeostasis [31]. Mfn2 is found not only on the OMM but also on the ER, and it acts as a tethering protein, bringing the ER and mitochondria into close proximity at specialized contact sites called MAMs [32]. These MAMs are crucial for efficient calcium exchange between the ER (a major intracellular calcium store) and mitochondria [33]. Mfn2 facilitates this calcium transfer, and alterations in Mfn2 levels or function can disrupt calcium signaling and contribute to mitochondrial dysfunction and cellular stress [34,35,36,37]. The interplay between calcium signaling and mitochondrial function is particularly critical in neurons [34].

**Cell Signaling:** Mitochondria participate in signaling, including apoptosis. They release cytochrome c, activating the caspase cascade [38]. Mitochondria are also a major source of ROS [39]. While high levels of ROS are detrimental, causing oxidative damage to cellular components (lipids, proteins, DNA), low levels of ROS act as signaling molecules [40]. These low levels of ROS, often referred to as “mitochondrial ROS” or “mitoROS”, can modulate various cellular processes, including gene expression, inflammation, autophagy, and cellular differentiation [40,41]. For example, mitoROS can activate transcription factors like NF-κB and HIF-1α, leading to changes in gene expression that help the cell adapt to stress [42]. They can also influence the activity of kinases and phosphatases, thereby modulating signaling pathways. However, when ROS production exceeds the cell’s antioxidant capacity, oxidative stress occurs, leading to cellular damage and contributing to disease pathogenesis [40,41].

**Metabolite Biosynthesis**: Mitochondria are crucial for the biosynthesis of a plethora of molecules and metabolites. This includes fatty acid and cholesterol synthesis, amino acid synthesis, and glucose and heme synthesis [43,44,45,46,47,48]. Mitochondria are central to fatty acid synthesis, providing the building blocks for membrane lipids [43]. They also play a role in the elongation and desaturation of fatty acids. Cholesterol synthesis, although primarily occurring in the ER, also involves mitochondrial enzymes [44]. The initial steps of the pathway, leading to the formation of mevalonate, occur in the cytosol, but subsequent steps, involving the conversion of mevalonate to squalene, occur in the ER and peroxisomes [44]. Mitochondria contribute to this process by providing precursors and energy. Mitochondria are involved in the synthesis of several amino acids, including glutamate, glutamine, aspartate, and alanine [45]. They participate in the interconversion of amino acids and their integration into metabolic pathways. Gluconeogenesis, the synthesis of glucose from non-carbohydrate precursors (like pyruvate, lactate, and glycerol), occurs partially in the mitochondria [45]. The enzyme pyruvate carboxylase, which catalyzes the first committed step in gluconeogenesis, is located exclusively in the mitochondrial matrix. Finally, heme synthesis, essential for hemoglobin and cytochromes, begins and ends in the mitochondria [47,48]. The first step (the condensation of glycine and succinyl-CoA to form δ-aminolevulinic acid, catalyzed by ALA synthase) and the final three steps of heme biosynthesis occur within the mitochondrial matrix [47,48].

### 2.2. Mitochondrial Quality Control Mechanisms

Several mechanisms maintain a healthy mitochondrial population:

**Protein Quality Control (Proteasome and Proteases):** The ubiquitin–proteasome system (UPS) degrades ubiquitinated proteins, including some mitochondrial proteins [49]. Inside mitochondria, proteases like Lon (matrix) and m-AAA/i-AAA proteases (IMM) degrade misfolded/damaged proteins [50].

**Mitochondrial-Derived Vesicles (MDVs):** MDVs are small vesicles (approximately 70–150 nm in diameter) that bud off from mitochondria [51]. They provide a mechanism for selectively removing damaged or oxidized mitochondrial components, including proteins and lipids, without requiring the degradation of the entire organelle. MDV formation can be triggered by oxidative stress, and the process appears to be distinct from mitophagy, although there may be some overlap in regulatory factors [52]. MDVs can carry specific cargo, suggesting a selective packaging mechanism. For example, some MDVs are enriched in oxidized mitochondrial proteins. These vesicles are ultimately delivered to lysosomes for degradation [51]. This is different from mitophagy, which generally involves the degradation of the entire mitochondrion. MDVs allow for the removal of damaged parts of mitochondria without sacrificing the whole organelle [53,54,55]. Furthermore, MDV formation can occur under basal, non-stressed conditions, whereas mitophagy is typically triggered by significant mitochondrial damage or depolarization [53]. The precise molecular mechanisms regulating MDV formation and cargo selection are still being investigated, but proteins involved in mitochondrial dynamics (like Drp1) and mitophagy (like PINK1 and Parkin) may play a role [52].

**Fission–Fusion Dynamics:** As described in Section 2.1, the dynamic balance between mitochondrial fission and fusion is essential for mitochondrial health. Fusion allows for the complementation of damaged mitochondria by mixing their contents with those of healthy mitochondria, diluting damaged components and restoring function [27,28,29]. Fission, on the other hand, segregates severely damaged portions of mitochondria, facilitating their removal via mitophagy [56]. This segregation prevents the spread of damage throughout the mitochondrial network [57]. During pathological conditions, this balance is often disrupted. In many neurodegenerative diseases, there is a shift towards increased mitochondrial fission and/or decreased fusion [57,58,59]. Excessive fission can lead to fragmented mitochondria, which are less efficient at ATP production, more prone to ROS generation, and may have impaired transport to distal neuronal processes [57,58,59]. Impaired fusion, on the other hand, can prevent the complementation of damaged mitochondria, leading to an accumulation of dysfunctional organelles [57,60,61]. Therefore, maintaining the proper balance between fission and fusion is crucial for mitochondrial quality control, and disruptions in this balance can contribute to disease pathogenesis [57,58,59,60,61].

**Mitophagy:** This selective autophagy removes damaged mitochondria. The PTEN-induced kinase 1 (PINK1)/Parkin pathway is well-studied. In damaged mitochondria, PINK1 accumulates on the OMM, recruiting Parkin, an E3 ubiquitin ligase. Parkin ubiquitinates OMM proteins, signaling autophagy receptors (p62/SQSTM1, NBR1, OPTN) to engulf the mitochondrion in an autophagosome, which fuses with a lysosome for degradation [14,62,63]. Other mitophagy pathways exist, including those mediated by BNIP3, Nix, and FUNDC1, which can act independently of PINK1 and Parkin.

## 3. Mitochondrial Dysfunction in Neurodegenerative Disease by Mechanism

One of the most consistently observed features of mitochondrial dysfunction in neurodegeneration is the excessive formation of ROS [64]. Under normal physiological conditions, a small proportion of electrons “leak” from complexes I and III of the ETC, generating superoxide (O_2_•^−^). This radical is rapidly converted to hydrogen peroxide by superoxide dismutase (SOD) isoforms, which is subsequently detoxified by catalase or glutathione peroxidase. However, in neurodegenerative conditions, this balance is frequently disrupted, leading to an overabundance of ROS that can oxidize lipids, proteins, and nucleic acids [65]. mtDNA, located in close proximity to the site of ROS generation and lacking protective mechanisms such as histones, is particularly vulnerable, resulting in mutations that further impair Adenosine Triphosphate (ATP) production [66].

In AD, Aβ peptides exacerbate ROS release by binding to mitochondrial proteins such as amyloid-beta-binding alcohol dehydrogenase (ABAD). In PD, dopamine metabolism within nigral neurons can spontaneously generate oxidative intermediates [67,68]. HD is associated with impaired activity in complexes II and III, increasing the risk of electron leakage, while ALS-linked mutations in SOD1 or TAR DNA-binding protein-43 (TDP-43) can disrupt normal ROS detoxification [69,70]. This ROS storm initiates a vicious cycle of damage to the ETC and mtDNA, exacerbating metabolic decline. Indeed, redox imbalance is a central feature in the pathophysiology of all four major neurodegenerative diseases.

Defective oxidative phosphorylation compromises ATP production, reducing the capacity of neurons to maintain ion gradients and potentially triggering excitotoxic cascades [71]. Studies in PD have demonstrated decreased complex I activity in the substantia nigra, correlating with the loss of dopaminergic neurons [72]. Environmental toxins targeting complex I, such as rotenone, replicate many clinical features of PD. In HD, multiple ETC complexes (particularly II–III) are impaired, leading to partial energy failure in striatal neurons [73]. AD brains exhibit diminished cytochrome c oxidase (complex IV) activity, while ALS is associated with broad ETC perturbations due to aggregated SOD1 or TDP-43 [74,75]. Even modest ETC dysfunction can result in subthreshold energy deficits that gradually erode neuronal integrity, particularly in aged brains with reduced metabolic resilience.

Furthermore, dysfunctional ETC complexes can leak electrons at an elevated rate, exacerbating oxidative stress [76]. This interplay between insufficient ATP generation and excessive ROS production underscores the pivotal role of mitochondria in neuronal viability. Notably, some therapeutic strategies aim to stabilize complex I or provide alternative energy substrates, although clinical success has thus far been limited [77,78].

As mentioned before, mitochondrial populations within neurons are maintained by a dynamic balance of fission (primarily mediated by Drp1) and fusion (Mfn1, Mfn2, and OPA1). Excessive fission produces small, fragmented mitochondria with reduced functional capacity and increased susceptibility to depolarization and ROS release [79]. Conversely, insufficient fission can result in elongated mitochondria that retain damaged segments. Research has shown that Drp1 hyperactivity is frequently observed in HD models, leading to a fragmented mitochondrial network in striatal neurons [80]. In AD, tau pathology may also contribute to aberrant Drp1 activation [81].

In PD, altered expression of Drp1 and Mfn2 can shift the mitochondrial network toward fragmentation. Over time, these morphological changes impair ATP delivery to synapses and hinder normal mitophagy [53]. Maintaining a balance between fusion and fission is essential for meeting local energy demands and segregating severely damaged organelles for clearance. Dysregulated mitochondrial dynamics thus sets the stage for neuronal stress and accelerates pathological cascades.

When mitochondria sustain damage or lose membrane potential, mitophagy pathways—particularly those mediated by PINK1 and Parkin—are responsible for their selective removal [82]. Failure of these mechanisms results in the accumulation of dysfunctional organelles that release ROS, proteotoxic fragments, and pro-apoptotic factors. PD-associated genes strongly implicate defective mitophagy, with mutations in PINK1 or Parkin reducing the ability to ubiquitinate and degrade damaged mitochondria [83]. Even in sporadic PD, disruptions in this pathway can exacerbate dopaminergic neuron loss.

Additionally, intramitochondrial proteases and chaperones maintain proteostasis by refolding or degrading misfolded proteins. In ALS, mutant SOD1 or TDP-43 can aggregate in the intermembrane space, impairing import channels and protease function [6,84]. In HD, mutant huntingtin can interfere with autophagic machinery, hindering the clearance of toxic protein fragments [85]. Thus, the quality-control axis—encompassing mitophagy, proteostasis, and mitochondrial import—emerges as a critical battleground in neurodegeneration, where disease-specific proteins disrupt normal clearance mechanisms.

A growing body of evidence links chronic neuroinflammation to mitochondrial decline in all major neurodegenerative diseases [86,87]. Damaged mitochondria release alarmins such as mtDNA, cytochrome c, and cardiolipin, which act as damage-associated molecular patterns (DAMPs) to activate microglia and astrocytes. Activated glial cells produce pro-inflammatory cytokines (e.g., TNF-α, IL-1β, IL-6) and reactive nitrogen species (e.g., nitric oxide), further compromising mitochondrial function [88]. Over time, a feedforward loop emerges; increased mitochondrial damage leads to higher DAMP release, intensifying inflammation and exacerbating neuronal stress.

In ALS, microglial activation often precedes overt motor neuron loss, suggesting an early interplay between innate immune responses and the metabolic demands of vulnerable neurons [89]. The role of chronic inflammation in PD, AD, and HD is also gaining recognition, particularly the concept that glial dysfunction amplifies mitochondrial stress, driving the progression from mild impairment to frank neuronal death [90].

Calcium overload in neurons can be lethal, and mitochondria play a critical role in buffering cytosolic Ca^2^⁺ during excitatory events [91]. At specialized contact sites between the ER and mitochondria, known as MAMs, local calcium transfer is tightly regulated. However, in conditions such as AD or ALS, disruptions in MAM structure promote excessive mitochondrial calcium uptake [13,92]. Elevated Ca^2^⁺ levels can open the mPTP, collapse membrane potential, and trigger apoptosis or necrosis.

**Figure 1:**
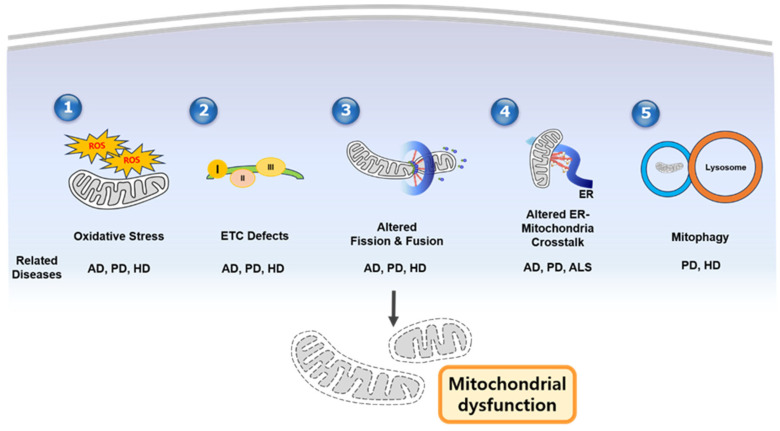
Schematic figure of mechanisms of mitochondrial dysfunction in neurodegenerative diseases. (1) Oxidative stress caused by excessive ROS, (2) ETC defects, dysregulated mitochondrial dynamics, (3) altered ER–mitochondrial crosstalk, (4) impaired mitophagy/quality control, and (5) mitophagy contribute to mitochondrial dysfunction. In addition, the middle part of the figure shows which disease is associated with each mechanism. AD—Alzheimer’s disease; PD—Parkinsons’ disease; HD—Huntington’s disease; ALS—amyotrophic lateral sclerosis.

PD may also involve altered ER–mitochondria coupling, exacerbating calcium imbalances and redox stress in dopaminergic neurons [93]. Notably, familial ALS mutations in TDP-43 or fused in sarcoma (FUS) impair normal protein handling at MAMs, highlighting how these subcellular junctions become vulnerable in disease states [6,94]. With excessive or prolonged calcium entry, mitochondria release apoptotic factors such as cytochrome c, culminating in irreversible neuronal loss (Figure 1).

## 4. Disease-Specific Mitochondrial Dysfunction

AD is the leading cause of dementia, clinically characterized by progressive cognitive decline and hallmark pathologies such as extracellular Aβ plaques and intracellular hyperphosphorylated tau tangles [94]. Numerous studies have identified early mitochondrial abnormalities in AD, including decreased cytochrome c oxidase (complex IV) activity, elevated ROS levels, and significant oxidative damage in brain tissue [95]. Aβ peptides not only disrupt synaptic function but also localize to mitochondrial membranes, where they inhibit key enzymes and exacerbate local free-radical production [96] (Table 1).

Tau pathology further exacerbates mitochondrial dysfunction by destabilizing microtubules, which are critical for axonal transport. This disruption prevents adequate mitochondrial distribution to dendrites and presynaptic terminals, impairing energy supply at these sites [124]. Additionally, tau can modulate Drp1, promoting excessive mitochondrial fission and fragmentation [51,125,126,127]. In terms of mitophagy, tau can impair mitophagy by inhibiting the translocation of Parkin to mitochondria [128]. Reduced expression of peroxisome proliferator-activated receptor gamma coactivator 1-alpha (PGC-1α), a master regulator of mitochondrial biogenesis, further limits the brain’s ability to replace damaged mitochondria [97]. Together, these factors create a vicious cycle of energy failure, oxidative stress, and synaptic deterioration that drives the clinical progression of AD. Despite extensive drug development targeting Aβ, the recognition of mitochondrial involvement in AD pathogenesis has spurred trials of antioxidant compounds and metabolic interventions, though success has been limited thus far [129].

While familial AD (fAD), caused by mutations in amyloid precursor protein (APP), presenilin 1 (PSEN1), or presenilin 2 (PSEN2), accounts for a small percentage of cases, the vast majority of AD cases are sporadic (sAD). Importantly, these mutations associated with fAD can directly impact mitochondrial function [98,99,100,101]. APP can interact with mitochondrial proteins and impair mitochondrial import and function [102]. Mutations in PSEN1/2, components of the γ-secretase complex, can alter Aβ production and directly affect mitochondrial calcium handling and ROS production [103,104]. In sAD, the APOE ε4 allele is a major genetic risk factor [105]. APOE4 impairs mitochondrial function, reduces mitochondrial respiration, and increases ROS production compared to other APOE isoforms [106].

PD primarily affects dopaminergic neurons in the substantia nigra pars compacta, leading to the cardinal motor symptoms of tremor, rigidity, and bradykinesia [7]. Mitochondrial dysfunction is deeply implicated in PD, as evidenced by early findings that the neurotoxin MPTP reproduces PD pathology by inhibiting complex I of the ETC [8,50]. Postmortem studies of PD tissue consistently reveal diminished complex I activity in the substantia nigra, correlating with elevated oxidative damage [130].

Genetic discoveries have further illuminated mitochondrial mechanisms in PD. Mutations in PINK1 or Parkin impair mitophagy, leading to the accumulation of defective mitochondria in dopaminergic neurons [14]. Deficits in DJ-1 compromise antioxidant defenses, while mutations in leucine-rich repeat kinase 2 (LRRK2) may disrupt cytoskeletal transport of mitochondria [107]. Additionally, α-synuclein aggregates can bind to mitochondrial membranes, exacerbating ETC deficits and intensifying ROS production [131]. Thus, PD exemplifies how gene–environment interactions converge on mitochondria, culminating in neuronal loss. Therapeutically, antioxidants, Drp1 inhibitors, and strategies to enhance PINK1–Parkin activity are under active investigation, although levodopa remains the gold standard for symptomatic treatment [132]. Familial PD can be caused by mutations in SNCA (α-synuclein), LRRK2, PARK2 (Parkin), PINK1, and PARK7 (DJ-1) [108,109,110,111,112]. Mutations in or overexpression of SNCA lead to α-synuclein accumulation, which directly interacts with mitochondrial membranes, impairing Complex I, increasing ROS, and disrupting dynamics [113]. The majority of PD cases are sporadic, influenced by environmental factors and aging [114]. Even without SNCA mutations, α-synuclein aggregation, a hallmark of PD, can impair mitochondrial function [113]. Increased oxidative stress and impaired antioxidant defenses also contribute to mitochondrial damage in sporadic PD [115].

As described before, HD is caused by an autosomal dominant expansion of CAG repeats in the HTT gene, leading to the production of mutant huntingtin protein with toxic polyglutamine tracts. The disease predominantly affects striatal medium spiny neurons [85]. Mitochondrial deficits manifest early in HD, with studies reporting compromised oxidative phosphorylation, morphological fragmentation, and abnormal calcium handling [116]. Mutant huntingtin interacts with transcriptional coactivators such as PGC-1α, impairing mitochondrial biogenesis and antioxidant responses [13]. Concurrently, overactivation of Drp1 drives excessive mitochondrial fission, fueling a cycle of ROS generation and bioenergetic collapse [80].

Striatal neurons rely on robust ATP production to manage excitatory glutamate inputs. When energy supply falters, excitotoxicity rapidly ensues, explaining the characteristic pattern of neuronal degeneration in the caudate and putamen observed in HD [117]. Therapeutic strategies have included creatine supplementation, triheptanoin, and small molecules that modulate mitochondrial fission. Gene-based approaches, such as antisense oligonucleotides to reduce mutant huntingtin expression, may indirectly improve mitochondrial function [118]. Although challenges remain, HD serves as a tractable model for studying how a single gene defect undermines mitochondrial integrity.

ALS is characterized by progressive muscle weakness and atrophy due to the selective degeneration of motor neurons in the spinal cord, brainstem, and motor cortex [6]. Approximately 5–10% of ALS cases are familial, with mutations in genes such as SOD1, TDP-43, and C9orf72, while the majority are sporadic, and have multifactorial origins [6]. Regardless of genetic status, mitochondrial pathology is a consistent feature, including morphological abnormalities, compromised ETC function, and protein aggregates localized to mitochondrial compartments [119]. TDP-43 pathology is common to both fALS and sALS, and TDP-43 can impair mitochondrial function [120,121,122,123].

Motor neurons, with their extensive axonal arbors, depend heavily on efficient mitochondrial transport to neuromuscular junctions for ATP supply. TDP-43 can form cytoplasmic aggregates that disrupt multiple cellular processes, including mitochondrial import. Mutant SOD1 can misfold in the mitochondrial intermembrane space, impairing respiration and catalyzing ROS production [133]. Additionally, astrocytes and microglia contribute to disease progression by releasing pro-inflammatory mediators, further heightening neuronal metabolic stress [89]. Currently, FDA-approved therapies such as riluzole and edaravone offer modest survival benefits. Advanced approaches aimed at restoring mitochondrial health—through genetic interventions, novel antioxidants, or corrected protein folding—are under intense investigation [134].

## 5. Therapeutic Strategies

Targeting mitochondrial dysfunction presents a promising, albeit challenging, therapeutic avenue for neurodegenerative diseases. Strategies aim to address various aspects of mitochondrial impairment, including oxidative stress, biogenesis, dynamics, calcium handling, and mitophagy (Table 2).

Given the central role of oxidative stress in neurodegeneration, exogenous antioxidants have been extensively explored to mitigate ROS-induced damage [16,26]. Compounds such as MitoQ, coenzyme Q10, and idebenone are designed to localize to mitochondria and scavenge free radicals at their source [135]. While preclinical studies have shown promise, these antioxidants have not consistently delivered robust clinical benefits, potentially due to challenges such as limited blood–brain barrier penetration, suboptimal dosing, or late-stage intervention [136].

Another approach focuses on enhancing the performance of the ETC. Small molecules that stabilize complex I or deliver alternative metabolic substrates, such as ketone bodies, aim to bypass glucose-based respiration and improve energy production [137]. Recently, boosting nicotinamide adenine dinucleotide⁺ (NAD⁺) levels through nicotinamide riboside or nicotinamide mononucleotide has gained traction for supporting cellular metabolism in models of AD, PD, and HD [138]. However, the success of such interventions depends on precise timing and targeted delivery to achieve meaningful neuroprotection.

Maintaining an optimal balance between mitochondrial fusion and fission is critical for protecting neurons from ROS escalation and facilitating the clearance of damaged organelles through mitophagy [79]. Excessive fission leads to mitochondrial fragmentation, which can be mitigated by inhibitors of Drp1, such as mdivi-1, or by upregulating Mfn2 to promote fusion. These strategies have shown potential in rescuing neuronal function in certain PD and HD models [53,139]. However, excessive fusion can also trap defective mitochondria within hyperfused networks, highlighting the need for fine-tuning mitochondrial dynamics. This could involve selectively modulating Drp1 phosphorylation or Mfn2 stability to achieve the desired balance [140].

The PINK1–Parkin pathway plays a central role in clearing damaged mitochondria, making it a key target for therapeutic intervention [14,82]. Pharmacological strategies to boost Parkin recruitment or mimic PINK1 phosphorylation have shown promise, particularly in PD models with mutations in these genes [141]. Similarly, improving the clearance of misfolded SOD1 in ALS may indirectly protect mitochondria [142]. Broadly, upregulating autophagic flux can help degrade aggregated proteins and defective organelles, though non-specific autophagy stimulation risks removing healthy cellular components [143]. The ideal approach likely involves targeting disease-specific cargo or augmenting selective mitophagy with minimal off-target effects.

Several compounds have been identified as potential mitophagy inducers, acting through various mechanisms [14,62]. Resveratrol, a natural polyphenol, found in grapes and red wine, has been shown to activate sirtuin 1 (SIRT1), a deacetylase that can promote mitophagy [144]. NADH and nicotinamide (NAM) are involved in cellular redox reactions and NAD+ metabolism. Increasing NAD+ levels, either directly with NADH or indirectly with NAM (a precursor), has been shown to enhance mitophagy, potentially through SIRT1 activation [144,145]. In terms of ubiquitin-specific Peptidase30 (USP30) inhibitors, USP30 is a deubiquitinase that opposes Parkin-mediated ubiquitination of mitochondrial proteins. Inhibiting USP30 can therefore promote mitophagy [145]. In terms of PINK1 activators, directly activating PINK1, the key initiator of the PINK1/Parkin mitophagy pathway, is another potential strategy. Some small molecules have been identified that can stabilize or activate PINK1 [145].

Gene-based approaches have garnered significant attention for treating familial neurodegenerative diseases, where single mutations (e.g., in HD, ALS, or PD) can be directly targeted [15]. Antisense oligonucleotides (ASOs) that reduce mutant huntingtin expression have advanced in clinical trials, partially restoring mitochondrial function in preclinical HD models [124]. Similar strategies aim to lower toxic SOD1 levels in ALS or deliver functional Parkin in PD [146]. CRISPR/Cas9 technologies are also being explored, though challenges such as safe delivery, immunogenicity, and editing efficiency remain. If these hurdles can be overcome, gene therapy may offer a disease-modifying option by preventing the production of proteins that harm mitochondria.

Lifestyle interventions, such as regular exercise, can elevate levels of PGC-1α, promoting mitochondrial biogenesis and supporting oxidative metabolism [147]. Dietary regimens like the ketogenic diet, which reduces reliance on glycolysis, have shown potential in moderating excitotoxic stress in HD and AD models [148]. Nutritional supplements, including creatine, L-carnitine, and alpha-lipoic acid, have also been tested, though outcomes have been mixed [149]. These low-risk interventions may complement pharmacological therapies, particularly when applied during prodromal or early disease stages.

Stem cell or induced pluripotent stem cell (iPSC)-derived neurons that supply trophic factors may help fortify mitochondrial health in AD or PD [150]. For example, dopamine neuron precursors transplanted into the putamen have shown potential in partially restoring dopaminergic signaling in PD, though long-term viability and functional integration remain variable [151]. Direct mitochondrial transplantation from healthy cells to injured ones is an emerging concept validated in some acute brain injury models. However, challenges such as immune response and efficient targeting limit its current utility in chronic neurodegeneration [152]. Overcoming these barriers could open new avenues for therapeutic rescue.

Despite the promising preclinical data for various mitochondrial-targeted therapies, translating these findings into clinically effective treatments for neurodegenerative diseases has proven challenging. Several clinical trials have been conducted, with mixed results. High-dose CoQ10 has been tested in PD, with some studies showing modest improvements in motor function or a slowing of disease progression, while others have shown no significant benefit compared to placebo [153,154,155,156,157]. Idebenone has shown some promise in Friedreich’s ataxia, a genetic mitochondrial disease, but its efficacy in AD and PD has been less conclusive [157,158,159,160]. Dichloroacetate (DCA), a pyruvate dehydrogenase kinase inhibitor that can increase flux through the pyruvate dehydrogenase complex and potentially improve mitochondrial function, has been tested in patients with congenital mitochondrial diseases and has shown some improvement in certain parameters, but its use in neurodegenerative diseases requires further investigation and careful consideration of potential side effects [161,162,163]. Mitophagy inducers, such as resveratrol, have also been investigated in several clinical trials [164,165,166]. Several factors contribute to the difficulties in translating preclinical success to the clinic. The heterogeneity of neurodegenerative diseases, with variations in genetic background, environmental exposures, and disease stage, makes it difficult to identify patient populations that will respond consistently to a given therapy. The blood–brain barrier (BBB) presents a significant obstacle, limiting the delivery of many potential drugs to the brain. The timing of intervention is also crucial; mitochondrial dysfunction may be an early event in disease pathogenesis, and therapies may be most effective if initiated before significant neuronal loss has occurred. Finally, the lack of sensitive and specific biomarkers to track mitochondrial function and treatment response in vivo makes it difficult to assess the efficacy of therapies in clinical trials.

## 6. Biomarkers and Future Directions

A critical need in neurodegenerative research is the identification of robust, noninvasive biomarkers to detect mitochondrial dysfunction at its earliest stages, when therapeutic intervention may be most effective [19,20,167]. Circulating mtDNA in plasma or cerebrospinal fluid shows promise as an indicator of neuronal mitochondrial damage or heightened cell turnover, though specificity concerns persist due to its elevation in other conditions [168]. mtDNA levels can also be measured in urine [169,170,171]. Measuring ETC enzyme activities in peripheral tissues, such as mononuclear cells or skin fibroblasts, may reveal deficits mirroring those in the central nervous system, though standardization of these assays remains incomplete [172]. Cytochrome c is normally located in the intermembrane space of mitochondria and plays a crucial role in the electron transport chain. Its release into the cytoplasm is a marker of mitochondrial damage and activation of apoptosis [173]. Cytochrome c levels can be measured in cerebrospinal fluid (CSF) and blood (Table 3).

In terms of markers of oxidative stress, oxidative stress leads to damage to various cellular components, including lipids, proteins, and DNA [174,175]. Products of lipid peroxidation (e.g., F2-isoprostanes, malondialdehyde), protein oxidation (e.g., protein carbonyls), and DNA oxidation (e.g., 8-hydroxy-2′-deoxyguanosine, 8-OHdG) can be measured in blood, CSF, and urine as indicators of oxidative stress and mitochondrial dysfunction [176,177,178].

Fibroblast growth factor 21 (FGF21) is a hormone secreted under mitochondrial stress and has been associated with cognitive decline in AD [179]. Growth differentiation factor 15 (GDF15) is similarly upregulated in response to mitochondrial dysfunction and may serve as a biomarker in PD [180]. While preliminary studies suggest that FGF21 and GDF15 may correlate with disease severity, additional large-cohort research is necessary to confirm sensitivity and specificity [180,181]. Urine is also emerging as a promising biofluid for detecting neurological changes. Recent reports highlight urinary metabolic profiles in AD or other disorders as a reflection of mitochondrial dysfunction, providing a convenient and noninvasive option for patient screening or longitudinal monitoring [169,170,171].

Metabolomics-based approaches can uncover signatures of lactate elevation or altered acylcarnitine profiles, potentially reflecting failing oxidative phosphorylation in preclinical or prodromal stages of AD, PD, and other conditions [182]. Neuroimaging strategies using specific positron emission tomography (PET) tracers to detect microglial activation or metabolic dysfunction are also in development, offering ways to localize brain regions most affected by mitochondrial decline in vivo, though correlation with actual mitochondrial health can be variable [183].

Efforts are underway to integrate multi-omics analyses—genomics, transcriptomics, proteomics, and metabolomics—to build personalized profiles of mitochondrial dysfunction and stratify patients who might respond best to targeted therapies [184,185,186,187]. Single-cell RNA sequencing of patient-derived neurons or glia can highlight distinct metabolic vulnerabilities in subpopulations of cells, providing refined insights into disease heterogeneity [185,186]. This personalization aligns with the idea that gene-specific interventions or combination treatments addressing oxidative stress, protein aggregation, inflammation, and mitochondrial imbalances simultaneously could yield significantly improved outcomes [150,188,189,190,191].

However, challenges remain in translating these advanced methods to routine clinical practice. Blood–brain barrier penetration and the timing of intervention are frequently cited as reasons why therapies successful in animals fail in humans [16,136,150]. Moreover, many current diagnostic criteria are met only after extensive neuronal loss has occurred. Early biomarker detection could shift the paradigm by identifying patients in prodromal or asymptomatic phases, enabling interventions that preserve neuronal function before irreversible damage sets in [8,183].

Another unresolved question is how to navigate the interplay between mitochondria and neuroinflammation. Disrupting the vicious cycle in which damaged mitochondria release DAMPs that fuel microglial activation—which in turn exacerbates oxidative stress—may require combined immunomodulatory and mitochondrial-protective therapies [86,88]. Further exploration of the endoplasmic reticulum–mitochondria interface may clarify how calcium dysregulation drives neurodegeneration in different disease contexts and could pinpoint additional molecular targets for novel drugs [13,92,131].

Progress in gene editing techniques, including refined CRISPR-based approaches, might eventually allow correction of monogenic forms of HD or familial ALS at earlier stages, halting the harmful cascade of mutant proteins that disrupt mitochondrial processes [93,118,146]. Although these technologies must overcome significant hurdles in safety, delivery, and off-target effects, their potential for truly disease-modifying interventions is immense.

## 7. Discussion

Mitochondrial dysfunction is a central and unifying feature across AD, PD, HD, and ALS. While these disorders exhibit distinct clinical phenotypes, they converge on overlapping mechanisms of neuronal injury, all of which revolve around the mitochondrion’s inability to meet high-energy demands, neutralize excessive ROS, and maintain protein quality control [7,9,14]. Research utilizing patient-derived cells, in vivo models, and postmortem analyses collectively highlights how imbalances in mitochondrial dynamics, impaired mitophagy, and defective ETC function create a “metabolic trap,” exacerbating neurotoxic pathways [16,70,139].

A key challenge lies in understanding the sequence of events in each disease: does mitochondrial dysfunction typically initiate subsequent protein aggregation and neuroinflammation, or does it primarily arise as a downstream consequence of toxic protein species? Evidence suggests a bidirectional relationship. For example, in PD, α-synuclein aggregates can localize to mitochondria, inhibiting ATP production and amplifying ROS [30,131]. Similarly, in AD, Aβ peptides disrupt mitochondrial function, while heightened oxidative stress accelerates the misfolding and oligomerization of proteins such as huntingtin in HD and SOD1 in ALS [38,133]. Once established, these feedback loops drive neurons toward irreversible decline unless interrupted by timely interventions.

Emerging therapeutic strategies reflect a growing sophistication in targeting mitochondrial dysfunction. The era of simple antioxidants dominating the therapeutic pipeline has given way to more precise approaches aimed at restoring mitochondrial networks, activating selective mitophagy, or modulating the complex interplay between mitochondria and glial cells [86,141]. Gene therapies targeting monogenic forms of HD or ALS may indirectly protect mitochondria by reducing the production of toxic proteins. Meanwhile, small molecules that recalibrate Drp1 activity hold promise for diseases characterized by excessive mitochondrial fragmentation. Lifestyle interventions, such as exercise and dietary modifications, though seemingly low-tech, engage potent endogenous pathways like PGC-1α–mediated mitochondrial biogenesis and warrant continued exploration [147,148].

Despite these advances, bridging the gap between promising laboratory findings and definitive clinical efficacy remains a formidable challenge. Timing is critical, as many patients present with advanced pathology where even near-complete restoration of mitochondrial function may not salvage heavily damaged neurons. Early detection efforts depend on the development of validated biomarkers capable of identifying subtle metabolic changes well before motor or cognitive symptoms manifest [19,20,168]. A major emphasis is also placed on multi-omics and single-cell analyses to capture the heterogeneous nature of these diseases and tailor interventions accordingly [185,186]. Recognizing that no single therapy is likely to succeed in isolation, many researchers advocate for combination approaches that address both mitochondrial deficits and co-occurring pathologies, such as inflammation and defective autophagy [84,87,183].

At the mechanistic level, further unraveling the coordination between mitochondria and other organelles, particularly the ER, may reveal critical nodes for therapeutic intervention [13,92]. Calcium overload at ER–mitochondria contact sites, known as MAMs, can directly drive neuronal death, suggesting that modulating these contact points could yield robust neuroprotection [131]. Additionally, clarifying the roles of newly identified mitochondrial stress responses, such as the mitochondrial unfolded protein response (UPRmt), may uncover novel pathways that neurons utilize to manage metabolic challenges [74,143]. Such insights could guide the development of targeted drugs that minimize systemic side effects while addressing specific mitochondrial vulnerabilities.

The role of neuroinflammation in exacerbating mitochondrial pathology is another frontier gaining increased attention [86]. Microglia and astrocytes, once viewed as mere bystanders or support cells, are now recognized as central modulators of neuronal fate. Future therapies might combine immunomodulators to suppress inflammatory cascades that damage mitochondria with small molecules that sustain ETC efficiency or facilitate the removal of defective organelles [150].

## 8. Conclusions

Mitochondrial dysfunction has emerged as a unifying thread in diverse neurodegenerative diseases, converging on interconnected mechanisms that drive neuronal injury. From oxidative stress and ETC deficits to dysregulated fission–fusion cycles and impaired mitophagy, mitochondrial collapse either initiates or exacerbates the pathological cascades observed in AD, PD, HD, and ALS. While no definitive cure has yet been realized, advances in gene therapy, selective autophagy modulation, and the precise regulation of mitochondrial dynamics offer promising glimpses of disease-modifying potential. Equally critical are innovations in biomarker development, which may enable earlier intervention at stages when compromised mitochondria can still be salvaged and neuronal networks remain partially intact. As our understanding deepens and multi-omics approaches refine personalized treatment strategies, the prospect of preserving or restoring mitochondrial health becomes increasingly tangible. Safeguarding the integrity of these organelles represents a central pillar in the ongoing effort to decelerate or potentially avert neurodegenerative decline.

## Figures and Tables

**Table 1:** Mitochondrial dysfunctions across major neurodegenerative diseases.

Disease	Central Mitochondrial Defects	Familial (Genetic) Mutations	Sporadic Factors	References
AD	Decreased complex IV activity, Aβ-induced ROS elevation, tau-driven transport failures, reduced PGC-1α expression, excessive fission	APP, PSEN1, PSEN2	APOE ε4, aging, inflammation	[1,9,10,12,81,97,98,99,100,101,102,103,104,105,106]
PD	Complex I inhibition, α-synuclein aggregates on mitochondrial membranes, impaired mitophagy (PINK1/Parkin), dopaminergic oxidative stress, possible Drp1/Mfn2 dysregulation	PINK1, Parkin, DJ-1, LRRK2	Environmental toxins (MPTP, rotenone), α-syn pathology	[2,7,8,14,72,107,108,109,110,111,112,113,114,115]
HD	Mutant huntingtin interfering with ETC (II–III), excessive Drp1-driven fission, calcium dysregulation, suppressed mitochondrial biogenesis via PGC-1α	HTT (CAG repeat)		[4,13,73,80,116,117,118]
ALS	Aggregated SOD1 or TDP-43 in mitochondrial compartments, decreased ETC function, severely compromised axonal transport to NMJs, glial-driven inflammatory loops	SOD1, TDP-43, C9orf72		[5,6,119,120,121,122,123]

AD—Alzheimer’s disease; PD—Parkinson’s disease; HD—Huntington’s disease; ALS—amyotrophic lateral sclerosis; Aβ—amyloid-β; ROS—reactive oxygen species; PGC-1α—peroxisome proliferator-activated receptor gamma coactivator 1-alpha; PINK1—PTEN-induced Kinase 1; Drp1—dynamin-related protein 1; Mfn2—MItofusin 2; ETC—electron transport chain; SOD—superoxide dismutase; TDP-43—TAR DNA-binding protein-43; APP—amyloid precursor protein; PSEN—presenilin; PINK1—PTEN-induced putative kinase 1; LRRK2—leucine-rich repeat kinase 2; HTT—huntingtin; APOE—apolipoprotein E; MPTP—1-methyl-4-phenyl-1,2,3,6-tetrahydropyridine.

**Table 2:** Therapeutic approaches targeting mitochondrial dysfunction.

Strategy Type	Examples/Compounds	Mechanism of Action	Potential Applications and Challenges	References
**Antioxidants and ETC Stabilizers**	Coenzyme Q10, MitoQ, NAD+ precursors	Scavenge ROS; enhance electron flow	Shown partial neuroprotection preclinically; BBB penetration and timing hamper clinical success	[8,16,26,135,136,137,138]
**Mitochondrial Dynamics Modulators**	mdivi-1 (Drp1 inhibitor), Mfn2 upregulators	Correct excessive fission; support fusion	Balancing is crucial; excessive fusion may trap damaged mitochondria	[53,79,80,139,140]
**Mitophagy Enhancers**	Parkin overexpression, resveratrol, NADH, NAM, USP30 inhibitors, PINK1 activators	Selective clearance of depolarized mitochondria	Strong rationale in PD with PINK1/Parkin loss; specificity needed to avoid unintended autophagic overload	[14,62,82,141,142,143,144,145]
**Gene Therapy**	ASOs for mutant huntingtin/SOD1; Lentiviral vectors for Parkin	Silence toxic genes or supplement protective ones	Delivery, stable expression, and immunogenicity remain barriers; especially relevant for monogenic forms	[5,15,146]
**Lifestyle and Nutritional Interventions**	Exercise, ketogenic diet, creatine	Bolster endogenous mitochondrial biogenesis, reduce ROS	Low risk but varied efficacy; synergy with pharmacological agents may yield best outcomes	[147,148,149]
**Cell and Mitochondrial Transplantation**	Stem cell–derived neurons, direct mito transfer	Replace dysfunctional neurons or supply healthy mitochondria	Limited by immune rejection, graft survival, and targeted delivery in chronic neurodegeneration	[150,151,152]

ETC—electron transport chain; NAD—nicotinamide adenine dinucleotide; Drp1—dynamin-related protein 1; Mfn2—MItofusin 2; NADH—nicoinamide adenine dinucleotide; NAM—nicotinamide; USP—ubiquitin-specific peptidase; PINK1—PTEN-induced kinase 1; ASOs—antisense oligonucleotides; SOD—superoxide dismutase; BBB—blood–brain barrier; PD—Parkinson’s disease.

**Table 3:** Biomarkers in mitochondrial dysfunction.

Biomarker	Biofluid(s)	Relevance to Neurodegenerative Diseases	Sensitivity/Specificity (If Known)	References
**mtDNA levels**	Blood, CSF, urine	Increased levels may indicate mitochondrial damage	Moderate sensitivity, limited specificity	[167,168,169,170,171]
**mtDNA mutations**	Blood, CSF, urine	Specific mutations may be associated with certain diseases	Variable, depending on the mutation	[167,168,169,170,171]
**Cytochrome c**	Blood, CSF	Marker of mitochondrial damage and apoptosis	Moderate sensitivity	[167,173]
**F2-isoprostanes**	Blood, CSF, urine	Marker of lipid peroxidation	Moderate sensitivity, limited specificity	[167,174,175]
**Protein carbonyls**	Blood, CSF, urine	Marker of protein oxidation	Moderate sensitivity, limited specificity	[137,174,175]
**8-OHdG**	Blood, CSF, urine	Marker of DNA oxidation	Moderate sensitivity, limited specificity	[167,176,177,178]
**FGF21**	Blood	Secreted under mitochondrial stress	May track disease progression; requires validation in larger cohort	[179,180]
**GDF15**	Blood	Upregulated by oxidative and mitochondrial stress	Good sensitivity but moderate specificity; more clinical data needed	[179,181]
**Urinary metabolites**	Urine	Reflect metabolic alterations in neurodegeneration	Under investigation, potential for non-invasive monitoring	[169,170,171]

mtDNA—mitochondrial DNA; 8-OHdG—8-hydroxy-2′-deoxyguanosine; FGF21—fibroblast growth factor 21; GDF15—growth differentiation factor 15; CSF—cerebrospinal fluid.

## Data Availability

No new data were created or analyzed in this study.

## Abbreviations

AD	Alzheimer’s disease
PD	Parkinson’s disease
HD	Huntington’s disease
ALS	amyotrophic lateral sclerosis
Aβ	amyloid-β
MPTP	1-methyl-4-phenyl-1,2,3,6-tetrahydropyridine
ETC	electron transport chain
ROS	reactive oxygen species
mtDNA	mitochondrial DNA
ER	endoplasmic reticulum
MAMs	mitochondria-associated membranes
OMM	outer mitochondrial membrane
IMM	inner mitochondrial membrane
IMS	intermembrane Space
OXPHOS	oxidative phosphorylation
NADH	nicotinamide adenine dinucleotide
FADH2	flavian adenine dinucleotide
ATP	adenosine triphosphate
ADP	adenosine diphosphate
Mfn1/2	MItofusin 1/2
OPA1	optic atrophy 1
Drp1	dynamin-related protein 1
Fis1	fission1
Mff	mitochondrial fission factor
MiD49	mitochondrial dynamics protein 49
MCU	mitochondrial calcium uniporter
mPTP	mitochondrial permeability transition pore
NCLX	Na^+^/Ca^2+^ exchanger
UPS	Uniquitin-proteasome System
MDVs	Mitochondrial-derived Vesicles
PINK1	PTEN-induced Kinase 1
SQSTM1	Sequestosome 1
NBR1	neighbor of BRCA1 gene
OPTN	optineurin
BNIP3	Bcl-2/adenovirus E1B 19 kDa-interacting protein 3
FUNDC1	FUN14 domain-containing 1
SOD	Superoxide Dismutase
ABAD	amyloid-beta-binding alcohol dehydrogenase
TDP-43	TAR DNA-binding protein-43
DAMPs	damage-associated molecular patterns
FUS	fused in sarcoma
PGC-1α	peroxisome proliferator-activated receptor gamma coactivator 1-alpha
fAD	familial AD
APP	amyloid precursor protein
PSEN	presenilin
sAD	sporadic AD
APOE	apolipoprotein E
LRRK2	leucine-rich repeat kinase 2
SNCA	α-synuclein
fALS/sALS	familial/sporadic ALS
NAD	nicotinamide adenine dinucleotide
SIRT1	sirtuin 1
NAM	nicotinamide
USP30	ubiquitin-specific Peptidase 30
DCA	dichloroacetate
BBB	blood–brain barrier
CSF	cerebrospinal fluid
8-OHdG	8-hydroxy-2′-deoxyguanosine
FGF21	fibroblast growth factor 21
GDF15	growth differentiation factor 15
PET	Positron Emission Tomography
CRISPR/Cas9	clustered regularly interspaced short palindromic repeats/CRISPR-associated protein 9
iPSC	induced pluripotent stem cell
UPRmt	mitochondrial unfolded protein response
